# Advances in Posteromedial Translation for Multiplanar Scoliosis Correction: Technique, Outcomes, and Future Directions

**DOI:** 10.7759/cureus.92928

**Published:** 2025-09-22

**Authors:** Phedy Phedy, Luthfi Gatam, Harmantya Mahadhipta, Asrafi R Gatam, Syafrudin Husin, Pranajaya D Kadar, Erica Kholinne

**Affiliations:** 1 Department of Orthopedics and Traumatology, Fatmawati Hospital, Jakarta, IDN; 2 Department of Orthopedics and Traumatology, Gatam Institute, Tangerang, IDN; 3 Department of Orthopedics and Traumatology, Premier Bintaro Hospital, Tangerang, IDN; 4 Department of Orthopedics and Traumatology, Faculty of Medicine, Universitas Sumatera Utara, Medan, IDN; 5 Department of Orthopedics and Traumatology, Faculty of Medicine, Universitas Trisakti, Jakarta, IDN

**Keywords:** biomechanics, pedicle screw, posteromedial translation, scoliosis surgery, spinal curvatures surgery

## Abstract

Posteromedial translation (PMT) is a widely utilized corrective technique in scoliosis surgery, offering a versatile approach to 3D deformity correction. Since its initial description, this technique has undergone significant evolution, incorporating various instrumentation systems such as sublaminar wires, polyester bands, hooks, claws, and pedicle screws. Posteromedial translation enables effective coronal correction, restoration of thoracic kyphosis, improvement of cervical lordosis, and rotational realignment, while minimizing stress on individual fixation points. Nevertheless, limitations remain, including neurological risks with sublaminar devices, implant-related complications, and varying efficacy in rotational correction. This comprehensive review examines the historical development, biomechanical principles, indications, instrumentation advancements, clinical outcomes, complications, and future perspectives of PMT in scoliosis surgery.

## Introduction and background

Severe scoliosis, characterized by an abnormal lateral curvature of the spine, often leads to significant deformities and clinical consequences such as pain, respiratory difficulties, and impaired mobility [1,2]. Traditionally, spinal deformities are corrected through posterior spinal fusion, a technique where screws and rods are used to stabilize the spine [3]. However, the surgical correction of scoliosis has undergone profound evolution over the past century, transforming from early nonoperative methods, such as bracing and in situ spinal fusions, into complex, highly technical procedures that address the entire 3D nature of spinal deformity [4,5]. Advances in spinal biomechanics, imaging technology, intraoperative navigation, and implant development have dramatically expanded the surgical armamentarium for managing scoliosis across all patient populations [4,6].

Posterior spinal surgery remains the cornerstone of most scoliosis corrections due to its versatility, safety, and ability to address deformities in all planes [7,8]. A wide range of posterior corrective strategies are now available, including global rod derotation, vertebral translation, cantilever bending, vertebral derotation, differential rod contouring, compression/distraction techniques, in situ contouring, and intraoperative traction-based methods. Each technique offers unique biomechanical advantages, with specific indications based on curve flexibility, magnitude, patient age, etiology, and associated comorbidities [9].

Among these various correction methods, posteromedial translation (PMT) has emerged as one of the most consistently utilized and adaptable techniques in modern scoliosis surgery [10]. Originally introduced as part of the early segmental spinal instrumentation era, PMT has undergone multiple refinements, transitioning from sublaminar wire systems to modern hybrid constructs incorporating pedicle screws, sublaminar bands, and reduction instruments [10,11]. The technique's controlled segmental correction enables the gradual, multiplanar realignment of complex spinal curvatures, with particular efficacy in restoring sagittal plane alignment, a key determinant of long-term functional outcomes [4,12,13].

Despite PMT’s widespread use and its inclusion in numerous instrumentation systems, a relative paucity of comprehensive, dedicated reviews remains that analyze its historical evolution, technical nuances, biomechanical rationale, clinical performance, and complications [10,14,15]. As surgical technology continues to advance, a clear understanding of PMT’s role within the broader context of scoliosis correction remains highly relevant for contemporary spine surgeons [16-18]. This review aims to comprehensively evaluate PMT as a technique, examining its biomechanical principles, historical development, instrumentation evolution, clinical outcomes, advantages, and limitations.

## Review

Methods

Study Design and Setting

**Figure 1:**
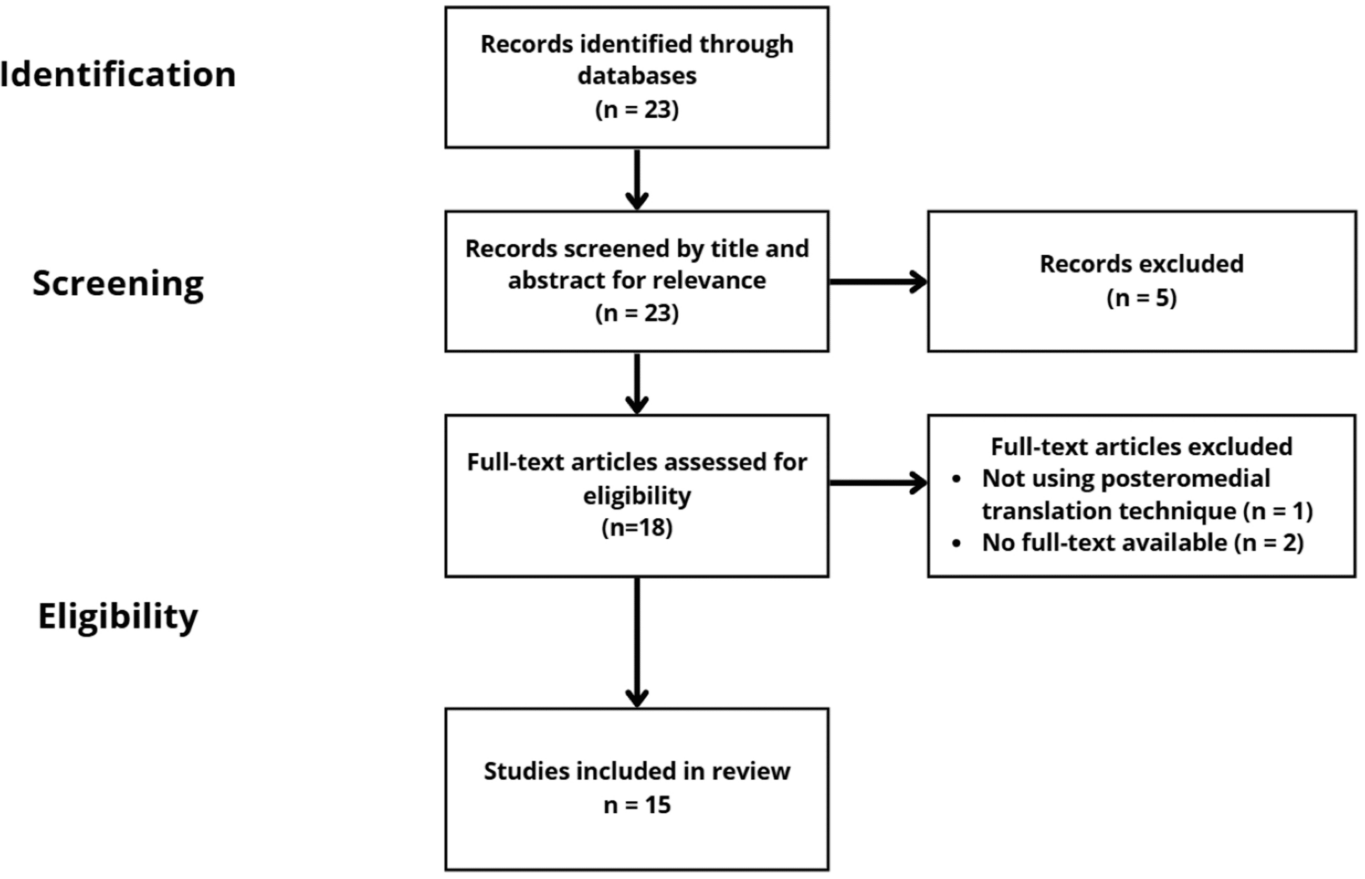
Study selection process

This study is a narrative review based on a search of academic databases. The literature search was conducted on Google Scholar and PubMed for articles dated up to June 30, 2025, using keywords such as “posteromedial translation” and “scoliosis.” The inclusion criteria specified studies published in English, peer-reviewed, and reporting on the biomechanics, historical background, instrumentation development, and clinical outcomes, as well as the advantages and limitations of PMT in scoliosis surgery. Eligible study designs included both prospective and retrospective investigations, as well as case series. Studies where no full text was available, non-English publications, and those that weren't peer-reviewed were excluded from the review. Studies were also excluded if they were expert opinions or editorial studies. A total of 23 records were identified through database searches. After screening titles and abstracts, 23 records were advanced to full-text review. Of these, 18 articles were assessed for eligibility. The full-text review excluded three articles because they did not use the PMT technique. Finally, 15 studies were included in the review (Figure 1).

Biomechanical principles of PMT

**Figure 2:**
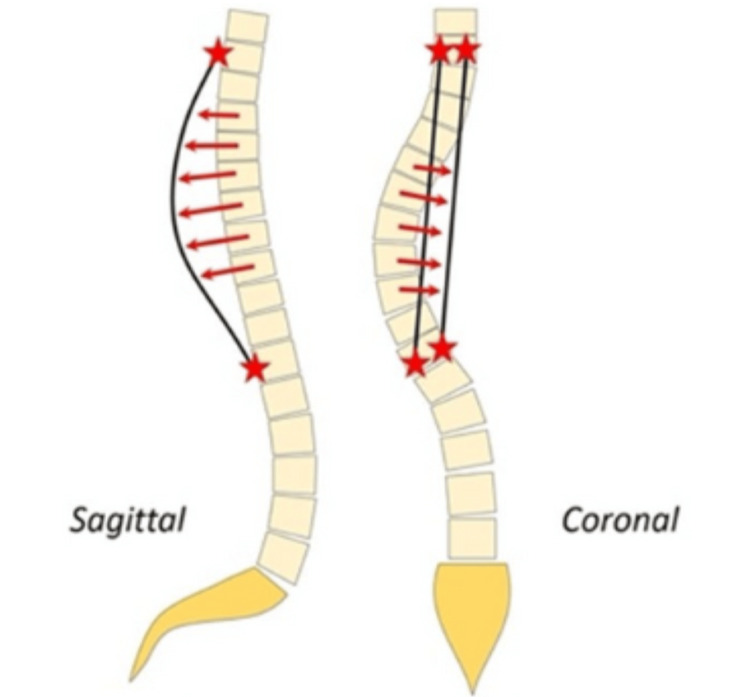
The schematization of the PMT technique PMT: Posteromedial translation Source: Reused with permission from *Comparison of Four Correction Techniques for Posterior Spinal Fusion in Adolescent Idiopathic Scoliosis, *Pesenti et al. [19].

The biomechanical principle behind PMT is grounded in addressing the multidimensional nature of spinal deformities seen in scoliosis (Figure 2) [19]. In scoliosis, the apical and peri-apical vertebral bodies are translated anteriorly and laterally relative to the midline, often accompanied by rotational deformity along the longitudinal axis of the spine [20]. This creates a complex 3D curvature that requires simultaneous correction across multiple planes. The PMT functions by applying targeted forces that draw these vertebrae posteriorly and medially, repositioning them in alignment with the pre-contoured rods, which are designed to mimic the physiological spinal curvature [10]. In the PMT technique, rod pre-contouring is typically performed to match the normal sagittal profile of the spine. However, in certain cases, it may be beneficial to introduce a more pronounced hyperkyphosis on the concave side. This adjustment is particularly useful when dealing with complex deformities, where greater correction is necessary. It is important to note that such hyperkyphosis should only be applied selectively, as part of a differential rod contouring strategy. Differential precontouring involves varying the degree of rod curvature based on the region of the spine, which can aid in achieving more precise deformity correction. This technique is especially valuable in patients with significant sagittal imbalance or those requiring specific segmental corrections. 

The process of correction in PMT typically begins with medial translation, which shifts the vertebral bodies toward the midline to restore coronal balance. Following medial translation, posterior translation is applied to reestablish appropriate sagittal alignment and restore thoracic kyphosis while avoiding the detrimental flattening effect often encountered with over-contoured rods [10,20]. The gradual and controlled nature of PMT allows for progressive correction, minimizing the risk of abrupt stress on spinal structures and reducing the likelihood of implant failure or neurological compromise [10].

Unlike some other corrective techniques that emphasize aggressive rod contouring or cantilever-based manipulation, PMT allows segment-by-segment alignment, providing fine control over both the magnitude and direction of corrective forces. This technique also permits dynamic adjustments during surgery to address variability in individual patient anatomy, curve flexibility, and intraoperative correction response [21]. Additionally, PMT's capability to correct deformity across coronal, sagittal, and axial planes while preserving vertebral rotation correction has contributed to its widespread adoption and ongoing refinement in modern scoliosis surgery [4,21-23].

Historical development of PMT

The evolution of PMT reflects the broader historical progress in segmental spinal instrumentation. Early attempts at scoliosis correction relied heavily on non-segmental constructs, often resulting in limited correction of deformity and higher rates of pseudarthrosis [10]. It was not until the late 1970s that Eduardo Luque revolutionized scoliosis surgery by introducing segmental sublaminar wire fixation. This technique involved inserting wires around the lamina of each vertebra, allowing for controlled, segment-by-segment translation of the spine toward pre-contoured rods. The Luque system provided unprecedented stability, improved correction rates, and superior maintenance of sagittal alignment. In his initial reports, Luque documented an average coronal correction of 72%, with a minimal loss of correction at follow-up, setting a new standard for scoliosis surgery [24]. However, while highly effective, Luque’s sublaminar wires posed challenges, particularly concerning neurological safety due to the passage of wires within the spinal canal. In response, subsequent developments sought to combine the corrective power of segmental instrumentation with safer anchorage methods [24,25].

In the 1980s and early 1990s, Asher introduced the Isola instrumentation system, which integrated pedicle screws, hooks, and sublaminar wires [26]. The hybrid configuration allowed for enhanced segmental control and reduced the need for sublaminar passage at every level. The Isola system became one of the first widely adopted hybrid constructs for scoliosis correction, demonstrating sustained long-term outcomes with stable deformity correction and high patient satisfaction rates [27].

Concurrently, Laxer introduced the Universal Spine System (USS), which incorporated powerful reduction tools such as the persuader device. In this system, the rods were locked into their final alignment early, and the persuader instrument was used to translate the spinal elements to the rods by gradually applying posterior-medial corrective forces. This approach represented one of the earliest devices to mechanize and standardize the application of PMT forces intraoperatively [28]. Further innovation emerged in the early 2000s with Mazda’s introduction of sublaminar bands and universal clamps. Replacing rigid stainless-steel wires with flexible polyester bands significantly reduced the risk of laminar fracture while eliminating concerns over galvanic corrosion between metallic components [10]. The greater contact surface area between the band and lamina allowed higher corrective forces while improving the safety profile during sublaminar passage. The universal clamp system demonstrated effective coronal, sagittal, and rotational corrections, further validating the versatility of PMT [10,11,14].

The introduction of segmental pedicle screw constructs, popularized by Suk in the mid-1990s, represented a significant paradigm shift in scoliosis surgery. Pedicle screws offered direct three-column spinal fixation, improving both coronal and axial plane correction while virtually eliminating implant failure [29,30]. The PMT principles are naturally adapted to pedicle screw constructs, especially with the development of reduction screws and extended-thread reduction sleeves, which permitted controlled translation of the spine toward the rod [30].

In the modern era, the combination of segmental pedicle screw constructs, sublaminar bands, hybrid fixation, and sophisticated reduction instrumentation has allowed surgeons to apply PMT with precision across a broad spectrum of scoliosis types and severities [20,23]. The technique continues to evolve with the integration of intraoperative navigation, neuromonitoring, and 3D imaging guidance, all of which contribute to enhanced safety, reproducibility, and correction outcomes [12,30].

Indications and patient selection

The PMT is a versatile corrective technique applicable across a broad spectrum of spinal deformities. While it has been most extensively studied in the treatment of scoliosis, its principles have also been successfully adapted to more complex pathologies. These include adolescent idiopathic scoliosis (AIS), neuromuscular scoliosis, congenital scoliosis, and adult spinal deformity [31]. The PMT may be particularly advantageous in patients with flexible curves, significant rotational deformity, or hypokyphotic thoracic spines where sagittal restoration is critical. In relation to the Lenke classification, the technique has been most frequently applied to thoracic-dominant patterns (Lenke types 1-4), where restoration of thoracic kyphosis is a primary surgical goal [12,32]. The procedure can be performed using sublaminar bands, hybrid constructs, or all-screw instrumentation, depending on the patient’s anatomy, bone quality, and the surgeon’s experience [10,13,33].

Clinical outcomes and efficacy

The clinical efficacy of PMT has been extensively reported across various implant configurations, patient populations, and curve types. A particular strength of PMT is its reproducible ability to restore both coronal and sagittal alignment while providing acceptable axial rotation correction, which together contribute to sustained long-term clinical outcomes [20,21].

Early Outcomes With Sublaminar Wires

In his pioneering work, Luque demonstrated the feasibility and efficacy of PMT using sublaminar wires. His series reported a mean coronal correction of 72%, with minimal loss of correction at the 18-month follow-up [24]. This represented a significant improvement compared to prior Harrington instrumentation, which was limited in its ability to restore sagittal balance and often resulted in flatback deformity [34]. Subsequent studies confirmed that segmental sublaminar constructs not only improved deformity correction but also enhanced spinal stability and reduced pseudarthrosis rates due to better load-sharing along the fusion mass [26,29].

Efficacy in 3D Correction

More recently, the 3D corrective power of PMT has been validated using advanced imaging modalities. Ilharreborde et al. conducted a multicenter study utilizing 3D imaging to assess PMT outcomes in AIS patients with hypokyphosis. Their results showed significant improvements in thoracic kyphosis (mean increase 8° ± 7°), though a subset of patients remained hypokyphotic postoperatively. Importantly, PMT demonstrated consistent coronal plane correction and partial restoration of vertebral rotation [20].

Sublaminar Bands vs. Pedicle Screws

The introduction of sublaminar bands provided an alternative anchorage system for PMT, particularly useful in patients with fragile or small pedicles [10]. Pesenti et al. compared sublaminar bands to pedicle screws and reported superior restoration of thoracic kyphosis in the band group, with a progressive improvement in kyphosis from 23.7° preoperatively to 34.0° at the two-year follow-up. However, the sublaminar band group demonstrated slightly inferior coronal plane correction compared to pedicle screw constructs (59.7% vs. 73%), and a minor loss of correction (~4°) was observed over time [22]. Despite these differences, the clinical outcomes in both groups were generally favorable, highlighting PMT's adaptability across fixation strategies.

Hybrid and Universal Clamp Systems

Hybrid systems, which combine sublaminar bands with hooks and screws, further enhance the versatility of PMT. Ilharreborde et al. demonstrated that universal clamp constructs achieved better overall correction across all planes compared to hook-only systems [23]. Moreover, hybrid PMT was associated with improvements in cervical alignment, with postoperative gains in cervical lordosis contributing to the restoration of global spinal balance, an increasingly recognized factor in long-term patient-reported outcomes in AIS [35].

The Frame Technique

One notable refinement of PMT is the frame technique, in which transverse connectors are temporarily affixed between bilateral rods during translation. This technique, introduced by Ilharreborde, enhances construct stiffness and helps preserve sagittal contour during correction. In their series, frame-assisted PMT produced superior medial translation and apical derotation (42.2% improvement in vertebral rotation), further demonstrating PMT’s capacity for comprehensive 3D deformity correction when combined with appropriate instrumentation [21].

Anchorage Site Modifications

Hirsch et al. studied alternative anchorage locations for band placement, comparing sublaminar versus sub-transverse process positioning. Both configurations achieved similar coronal and sagittal plane corrections. However, the sub-transverse process approach offered a potential safety advantage by avoiding the spinal canal altogether, thereby reducing the risk of neurologic complications associated with traditional sublaminar passage [33].

Influence of the Rod Material

The type of rod material used during PMT can also influence outcomes. Titanium alloy rods offer greater flexibility, whereas cobalt-chromium rods provide higher stiffness and superior maintenance of sagittal correction. Angelliaume et al. demonstrated that cobalt-chromium rods achieved better kyphosis restoration in hypokyphotic patients undergoing PMT, particularly when hybrid constructs were used [18]. However, other studies have shown that with high-density pedicle screw constructs, both titanium and cobalt-chromium rods produce comparable coronal correction, suggesting that rod material selection should be individualized based on the primary correction goals [36].

Long-Term Stability

Available long-term follow-up data suggest that PMT offers durable correction with low rates of hardware failure and revision surgery when applied appropriately. The segmental nature of PMT provides excellent load sharing, promoting solid fusion and minimizing junctional problems [24]. While some studies report minor correction loss over time (typically within two to four degrees), this is generally not clinically significant and does not impact patient satisfaction or functional outcomes [12,13,33,35].

Advantages and limitations

Several comparative studies highlight the advantages of PMT over other correction techniques. In a multicenter cohort of 562 AIS patients, PMT resulted in a +16° gain in thoracic kyphosis, compared with +7° using rod rotation and -5° with in situ bending or cantilever techniques [19]. The PMT provides strong sagittal correction, particularly for thoracic kyphosis, while offering controlled, segmental correction across multiple planes. Its flexibility allows the use of sublaminar bands, pedicle screws, and hybrid constructs, often minimizing the need for aggressive rod contouring [10,20]. Compared with the original Luque technique, which relied on blind passage of multiple sublaminar wires and carried a higher risk of cord or root injury, modern PMT instrumentation offers significant safety advantages. The use of sublaminar bands, pedicle screw fixation, advanced imaging, and intraoperative neuromonitoring has markedly lowered neurological risk. Nevertheless, some risk remains in PMT due to the close relationship of neural structures during sublaminar passage and translation [33].

Some studies have reported complications associated with PMT, including superficial skin infection (5.3%) and neurological deficit (0.8%) [11,37]. Rotational correction may be limited, and sublaminar bands offer lower pullout strength than pedicle screws [26]. Some cases may require longer fusions, and rare complications like granuloma formation have been reported, underscoring the need for careful surgical planning [14,23]. The summary of the advantages and limitations of PMT can be seen in Table 1.

**Table 1:** The advantages, limitations, and complications of PMT PMT: Posteromedial translation

Advantages	Limitations and complications
Strong sagittal plane restoration, particularly kyphosis correction	Neurologic risks (e.g., paresthesias, weakness, canal compromise)
Segmental, controlled correction minimizes the risk of overcorrection or abrupt loading	Lower rotational correction potential due to posterior anchor position
Compatibility with both sublaminar bands and all-screw constructs	Reduced correction force (lower pullout strength of sublaminar bands compared to pedicle screws)
Reduced need for aggressive rod contouring compared to differential rod contouring	Increased fusion length may be required for optimal correction
Multiplanar correction is achievable with frame and hybrid techniques	Rare implant-related complications (e.g., granuloma formation with universal clamps)

Future directions

Continued research into optimized construct configurations, hybrid anchorage techniques, rod material properties, and computer-assisted navigation may enhance the safety and effectiveness of PMT. Emerging 3D imaging technologies (electro-optical system (EOS), CT-based analysis) may improve intraoperative precision and postoperative assessment of correction. Further prospective, randomized studies are needed to directly compare PMT with other modern techniques, such as global derotation, cantilever correction, and differential rod contouring, in various scoliosis subtypes.

## Conclusions

Posteromedial translation remains a cornerstone technique in scoliosis surgery, offering controlled, multiplanar correction with particular strength in restoring thoracic kyphosis. The evolution of sublaminar bands, pedicle screws, hybrid constructs, and reduction devices has broadened its application and improved its safety profile. Nevertheless, careful consideration of patient selection, implant choice, and technical execution is critical to maximizing outcomes while minimizing risks. Ongoing advancements in surgical technology and instrumentation will continue to refine the role of PMT in contemporary scoliosis correction.
